# Computational and Experimental Evaluation of the Immune Response of Neoantigens for Personalized Vaccine Design

**DOI:** 10.3390/ijms24109024

**Published:** 2023-05-19

**Authors:** Iker Malaina, Lorena Gonzalez-Melero, Luis Martínez, Aiala Salvador, Ana Sanchez-Diez, Aintzane Asumendi, Javier Margareto, Jose Carrasco-Pujante, Leire Legarreta, María Asunción García, Martín Blas Pérez-Pinilla, Rosa Izu, Ildefonso Martínez de la Fuente, Manoli Igartua, Santos Alonso, Rosa Maria Hernandez, María Dolores Boyano

**Affiliations:** 1 Department of Mathematics, Faculty of Science and Technology, University of the Basque Country (UPV/EHU), 48940 Leioa, Spain; 2 NanoBioCel Research Group, Laboratory of Pharmaceutics, School of Pharmacy, University of the Basque Country (UPV/EHU), 01006 Vitoria-Gasteiz, Spainrosa.hernandez@ehu.eus (R.M.H.); 3 Bioaraba, NanoBioCel Research Group, 01009 Vitoria-Gasteiz, Spain; 4 Luis Martínez, Basque Center for Applied Mathematics BCAM, 48009 Bilbao, Spain; 5 Biomedical Research Networking Centre in Bioengineering, Biomaterials and Nanomedicine (CIBER-BBN). Institute of Health Carlos III, 28029 Madrid, Spain; 6 Department of Dermatology, Basurto University Hospital, 48013 Bilbao, Spain; 7 Biocruces Bizkaia Health Research Institute, 48903 Barakaldo, Spainlola.boyano@ehu.eus (M.D.B.); 8 Department of Cell Biology and Histology, Faculty of Medicine and Nursing, University of the Basque Country (UPV/EHU), 48940 Leioa, Spain; 9 Technological Services Division, Health and Quality of Life, TECNALIA, 01510 Miñano, Spain; 10 CEBAS-CSIC Institute, Department of Nutrition, 30100 Murcia, Spain; 11 Department of Genetics, Physical Anthropology and Animal Physiology, Faculty of Science and Technology, University of the Basque Country (UPV/EHU), 48940 Leioa, Spain

**Keywords:** bioinformatics, ex vivo, human leucocytic antigen, immunogenicity, nanoparticle, neoantigen, vaccine design

## Abstract

In the last few years, the importance of neoantigens in the development of personalized antitumor vaccines has increased remarkably. In order to study whether bioinformatic tools are effective in detecting neoantigens that generate an immune response, DNA samples from patients with cutaneous melanoma in different stages were obtained, resulting in a total of 6048 potential neoantigens gathered. Thereafter, the immunological responses generated by some of those neoantigens ex vivo were tested, using a vaccine designed by a new optimization approach and encapsulated in nanoparticles. Our bioinformatic analysis indicated that no differences were found between the number of neoantigens and that of non-mutated sequences detected as potential binders by IEDB tools. However, those tools were able to highlight neoantigens over non-mutated peptides in HLA-II recognition (*p*-value 0.03). However, neither HLA-I binding affinity (*p*-value 0.08) nor Class I immunogenicity values (*p*-value 0.96) indicated significant differences for the latter parameters. Subsequently, the new vaccine, using aggregative functions and combinatorial optimization, was designed. The six best neoantigens were selected and formulated into two nanoparticles, with which the immune response ex vivo was evaluated, demonstrating a specific activation of the immune response. This study reinforces the use of bioinformatic tools in vaccine development, as their usefulness is proven both in silico and ex vivo.

## 1. Introduction

In recent years, personalized antitumoral vaccination has been increasingly welcomed as an innovative and promising approach to treating several types of cancers [1,2,3,4]. The key reason for choosing personalized vaccination against cancer cells is that tumors contain a large number of mutations, and approximately 95% of these mutations seem to be unique to that tumor [5]. Thus, these mutations constitute ideal oncological targets for efficiently targeting individual tumors [6], particularly for personalized vaccination.

However, although the number of mutations in tumors is considerable, in order to create an effective vaccine, the first step is to distinguish between mutations only present in the tumor, and those that occur in the remainder of our non-oncogenic cells. This is where the concept of neoantigens arise, which are members of a class of peptides that arise from tumor-specific mutations and bind to the Major Histocompatibility Complex (MHC). In humans, this complex is denoted as HLA, referring to the Human Leucocyte Antigen [7]. These neoantigens have not been previously challenged by the immune system, and, consequently, the immune system will not apply tolerance mechanisms against them [8].

Nevertheless, although targeting neoantigens has resulted in clinical benefits [9,10], if the whole mutation spectrum of a tumor (known as a mutanome) is considered, the number of potential neoantigens is vast. Thus, blindly selecting candidates does not ensure inducing highly immunogenic responses. Generating an immune response against a mutated peptide depends directly on the patient’s HLA system’s capacity to bind the neoantigen and present it to lymphocytes [11]. Therefore, selecting those antigens that bind more effectively to the immune system’s cells is a reasonable first criterion for neoantigen-based vaccine design.

With this purpose, and since the individual evaluation of every neoantigen in a tumor is too expensive and time-consuming, several bioinformatic tools for the in silico prediction of Class I immunogenicity, HLA-I and HLA-II binding affinity, TAP transport, etc., have already been developed [12,13,14,15]. Before applying these tools to experimental data, it would be necessary to evaluate whether these bioinformatic techniques can effectively distinguish potential neoantigens from non-mutated versions based on their estimated immunological characteristics. Moreover, it would also be important to determine whether the experimental results can confirm the effectiveness of these tools in developing efficient personalized vaccines.

First, with the aim of providing a response to these issues, portions of the mutanomes from six patients with cutaneous melanoma were sequenced. This type of skin cancer is located in the epidermis and arises from pigment-containing cells called melanocytes [16]. It is highly invasive and metastatic [17] and has a high mutation rate, making it an excellent candidate for addressing our problem.

After identifying the amino acid sequence of the peptides corresponding to the detected DNA mutations, the predicted Class I immunogenicity and HLA binding affinity of potential neoantigens and their respective non-mutated versions were studied with the IEDB prediction tool [13]. An exhaustive analysis of 6048 mutated sequences (peptides obtained from the mutated sequence, which were considered as potential neoantigens) and their respective non-mutated versions (peptide sequences without the mutation that originated in the tumor) was performed. Using this information, the in silico results of both groups were compared, and the result answered the first question mentioned above, i.e., whether the estimation of the generated immunogenic response for neoantigens is greater than that for the non-mutated versions, according to bioinformatic tools.

Thereafter, in order to experimentally validate the use of these predictive tools, a vaccine candidate against cutaneous melanoma, based on these bioinformatic methods, was developed and tested. Thus far, the usual process for selecting the best neoantigens for vaccine development against melanoma has involved employing stable sorting algorithms [3]. In contrast, in this study, aggregative functions were used because a set of solutions in the Pareto front, which had a more balanced trade-off between the objective functions compared to lexicographic orders, was obtained. Finally, the vaccine was synthesized and entrapped in polyethylenimine (PEI)-coated poly-lactic-co-glycolic acid (PLGA) nanoparticles (NPs) and tested for effectiveness and specificity ex vivo, thereby answering the second question of the study.

In this work, for the first time, a two-fold validation for the use of bioinformatic tools in personalized vaccine development is presented, one computational and the other experimental. For this validation, more than 6000 potential neoantigens were evaluated using widely used bioinformatics tools offered by IEDB. These peptide sequences, instead of being simulated, were obtained from experimental data of melanoma patients, and in order to be evaluated, their specific corresponding HLA molecules were considered. In the second segment, combinatorial optimization was applied to outperform previous methods for melanoma vaccine design (which usually applied lexicographic ordinations) by introducing a novel target function that combines the seven considered properties. Regarding the ex vivo validation portion, studies evaluating nanoparticulate neoantigens have typically employed murine models [18,19,20,21]. However, investigating these neoantigens with a patient’s own cells enables us to determine whether the predicted neoantigens elicit an immune response in that specific patient, and whether this response is enhanced by nanoparticle delivery. On the other hand, despite that PLGA NPs have already been studied as antigen carriers for cancer treatment [22], the in silico neoantigen determination has been added, instead of only cancer cell lysates that dilute interesting antigens with many cell compounds.

Our study encourages the use of bioinformatic predictions for neoantigen selection, which, combined with the knowledge of immunologists, could improve vaccine efficiency, one of society’s main interests today.

## 2. Results

The results have been divided into two sections. Firstly, the immunological estimation of neoantigens and non-mutated strings was compared using bioinformatic tools. Secondly, whether predicted neoantigens selected for their estimated immunological characteristics could trigger an effective immune response following encapsulation into NPs was tested. In other words, their efficacy as a personalized vaccine was experimentally tested.

### 2.1. Computational Analysis of Immunological Characteristics

**Figure 1:**
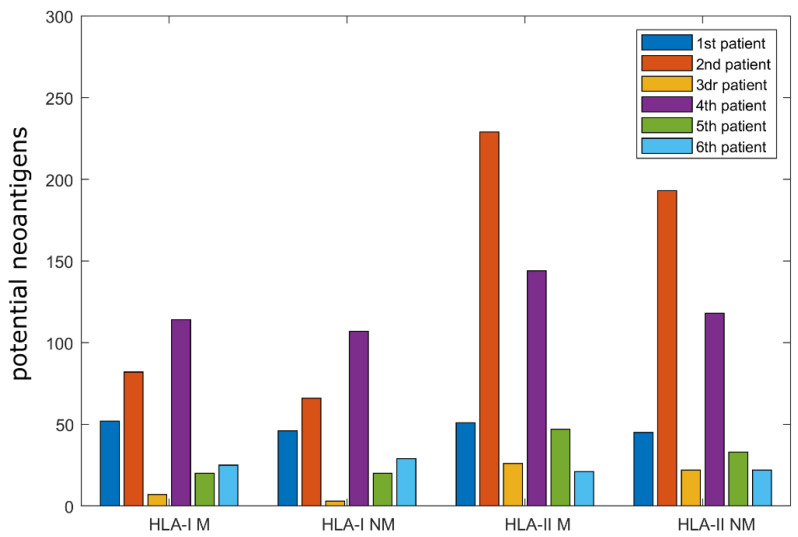
Bar graph illustrating the number of peptides that passed the respective cutoff points (≤1% for HLA-I and ≤10% for HLA-II binding affinity) for mutated (M) and non-mutated (NM) peptides.

**Figure 2:**
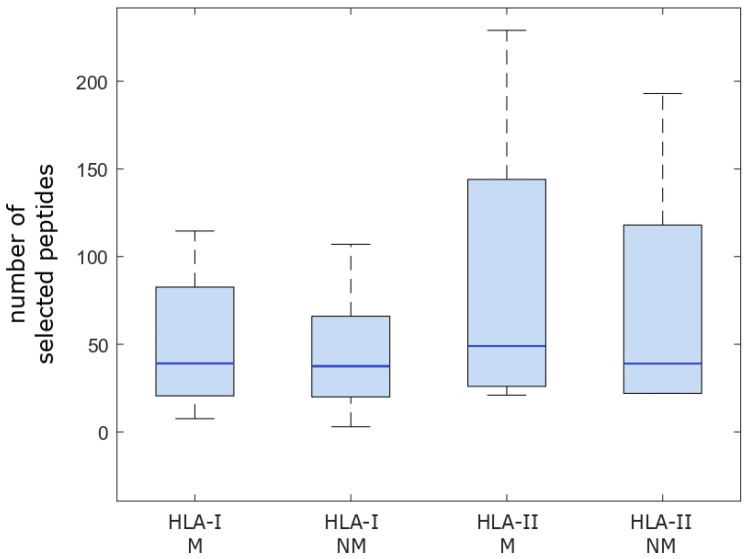
Box plot illustrating the distributions of the number of peptides that passed the respective cutoff points (≤1% for HLA-I and ≤10% for HLA-II binding affinity). M indicates mutated peptides, while NM references the non-mutated versions. The light blue boxes represent the distribution of the central 50% of the values and the dark blue lines represent the medians. The remainder of the values are represented by the arms.

In order to compare the immunological properties of potential neoantigens and their non-mutated versions computationally, three main variables were estimated: MHC Class I and II binding affinities, and Class I immunogenicity using IEDB’s bioinformatic tools [13], as explained in the Materials and Methods, Section 4. The mutation was positioned at the center of the neoantigen peptide, meaning that, for antigens of length 17 (considered in the first segment of the study), the mutation was placed at the 9th amino acid (for further details, see Materials and Methods, Section 4.1.3). First, in order to achieve this, the HLA alleles of the patients, which are depicted in Table 1 (for HLA-I) and Table 2 (for HLA-II), respectively (for more information see Section 4.1.1. and Section 4.1.2), were obtained:

First, the estimations of binding affinities were studied, following the recommendations from IEDB. In these cases, the “percentile rank” variable was used to filter potential binders from those that predictably would not be good binders. In order to cover most immunological peptides, IEDB recommends selecting strings with a “percentile rank” ≤ 1% for MHC Class I [23,24], while for MHC Class II, the recommended “percentile rank” is ≤10%. Figure 1 displays the number of potential HLA-I and HLA-II binders by patient, while Figure 2 illustrates the distribution of these values in a box plot.

In order to provide a wider picture of the peptides fulfilling this criterion, the relative percentages of each group were calculated. Among the considered strings, the percentage of mutated peptides that would potentially bind HLA-I molecules was 0.8% ± 0.38% (average ± standard deviation), ranging from 0.3% to 1.2%. For their non-mutated versions, the results were 0.75% ± 0.46%, ranging from 0.13% to 1.4%. When HLA-II was analyzed, the mutated peptides were 15.24% ± 2.96%, ranging from 10.63% to 19.88%. Finally, the non-mutated versions were 13.02% ± 2.93%, ranging from 9.38% to 16.75%.

In order to compare the data presented in Figure 2, which illustrates the distributions of the number of peptides that passed the threshold grouped by HLA-I or II, mutated or non-mutated, whether the variables were normally distributed was assessed first. The *p*-values for the tests were 0.53, 0.57, 0.43, and 0.48 for HLA-I mutated, HLA-I non-mutated, HLA-II mutated, and HLA-II non-mutated peptides, respectively. Therefore, in order to compare the distributions, independent *t*-tests were performed (note that below, the paired case was analyzed, distinguished by patient). For the HLA-I group, the *p*-value was 0.841, the confidence interval (CI) was (−45.93, 55.26), and the *t*-statistic was 0.205. For the HLA-II group, the *p*-value was 0.748, the confidence interval was (−83.59, 112.59), and the *t*-statistic was 0.329. Thus, no significant differences were found when comparing the distributions of the number of peptides that passed the threshold as a group (for more details, see Section 4.1.4).

Subsequently, in order to determine whether the mutated versions of peptides exhibit more binding affinity than non-mutated versions for each patient, two comparisons were performed: Firstly, the number of predicted HLA-I mutated peptides to the number of non-mutated peptides was compared. Secondly, the number of predicted HLA-II mutated peptides to the number of non-mutated peptides was compared. Since normality was assumed in all cases, a paired *t*-test was performed for each HLA couple (i.e., number of mutated versus non-mutated peptides for each patient).

The alternative hypothesis was that the difference between the average of mutated potential neoantigens and non-mutated ones was greater than 0. With a significance level of 5%, the paired *t*-test yielded a *p*-value of 0.08, a confidence interval of $CI=\left(-1.50,\infty \right)$, and a *t*-statistic of 1.6. Therefore, no significant differences were found between the number of mutated and non-mutated peptides that potentially bind HLA-I molecules.

For the number of HLA-II peptides, the *p*-value was 0.03, the confidence interval was $CI=\left(2.44,\infty \right)$, and the *t*-statistic was 2.43. These results indicate that mutated peptides are more likely to pass the detection threshold of HLA-II molecules than non-mutated ones, according to these bioinformatic tools.

Second, the Class I immunogenicity prediction values for both groups were evaluated. In particular, the alternative hypothesis studied was that the mutated strings presented higher Class I immunogenicity than the non-mutated versions, with a significance of 5%. In contrast to the previous comparison, the analysis for this statistic was different, since here, instead of being a “yes/no” question, since it measures potential binding capability, the probability of generating an immune response is incremented as the variable increases. The *p*-value for this comparison was 0.96, the confidence interval was $CI=\left(-0.03,\;\infty \right)$ and the *t*-statistic was −1.78. Therefore, no significant increase in the immunogenicity values of neoantigens was detected with respect to non-mutated strings.

### 2.2. Ex Vivo Validation of the Immune Response

#### 2.2.1. Optimization of Neoantigen Selection for Vaccine Design

For the second segment of the study, in which the objective was to test the capacity of our neoantigens to generate an immune response ex vivo, a vaccine using an optimization technique applied to aggregative functions was designed first. Before applying the optimization technique described in the Materials and Methods, Section 4 to select the best neoantigens for the vaccine, additional bioinformatic characteristics, aside from the three main variables mentioned in the first segment of the study (Class I immunogenicity and HLA-I and II binding affinities), were estimated. These variables were included to enhance the efficiency of the potential vaccine candidate and complement the aforementioned variables, which are more related to immunogenicity. Specifically, the hydrophobicity/hydrophilicity through the GRAVY index score (to maximize neoantigens on the surface [25,26,27]), the proteasome cleavage/TAP transport (to improve the affinity prediction of neoantigens [28]), the VaxiJen score (to select those with a higher probability of being recognized as tumor antigens [29]), and the frequency of the mutation (to maximize neoantigens containing the most frequent mutations) were calculated. For more information about these variables and their estimation, please refer to the Materials and Methods, Section 4.

Thereafter, the six neoantigens that maximized the weighted sum of the seven variables from the set of all neoantigen candidates (represented in Table S1) were selected, according to the optimization method and weighting procedure proposed in the Materials and Methods, Section 4. In short, the goal of the optimization is to obtain the most promising combination of the selected characteristics. Thus, for each neoantigen n, the amount to be maximized was:

$$f\left(n\right)=0.2\;Nimm_{i}\left(n\right)+0.2\;NHLAI_{i}\left(n\right)+0.2\;NHLAII_{i}\left(n\right)+0.1\;Nvf_{i}\left(n\right)+0.1\;Nap_{i}\left(n\right)+0.1\;Nhpl_{i}\left(n\right)+0.1\;NTAP_{i}\left(n\right).$$

Since a set of six neoantigens was used for the experimental validation, the final objective function was $F\left(S\right)=\underset{n\in S}{\sum }f\left(n\right)$, where S is a subset of cardinality six of the set N of neoantigens (for more information about the optimization, see Materials and Methods, Section 4.2.3).

As can be observed, the main variables (Class I immunogenicity, HLA-I binding, and HLA-II binding) were considered with greater weight, with respect to the other variables, to highlight their influence, since the objective of this study was to evaluate them, in particular. The results of the variables for the selected neoantigens through our optimization method are depicted in Table 3. It is worth noting that all of the selected neoantigens are 15 amino acids long.

Note that all of the neoantigens in Table 3 (with the exceptions of MRHSFFSEVNWQDVY for HLA-I and FRDQSLSYHHTMVVQ for HLA-II) obtained positive values for HLA binding for both classes, indicating that these peptides would likely be good binders for the corresponding patient’s HLA molecules. However, it is important to note that binding affinity alone does not guarantee immunogenicity or the ability to induce a strong immune response.

Subsequently, these six neoantigens were grouped in the following two peptides with lengths of 45 (with three consecutive peptides each):

Peptide 1: DWLEWLRQLSLELLKFRDQSLSYHHTMVVQIGRFANYFRNLLPSN
Peptide 2: MRHSFFSEVNWQDVYRLFMHHVFLEPITCVCSRRFYQFTKLLDSV

The peptides were synthesized, and then encapsulated into two types of nanoparticles, as described below.

#### 2.2.2. Optimization of Neoantigen Selection for Vaccine Design

The nanoparticles (NPs) were prepared using the w/o/w method, and were analyzed using the dynamic light-scattering technique. The size of the PEI-coated PLGA NPs ranged from 280 to 350 nm, with a narrow size distribution, as demonstrated by the low polydispersity index (PdI). The surface charge was confirmed to be positive (around 40 mV), due to the PEI-coating, by ζ-potential analysis (refer to Table S2 for details).

The amount of neoantigen encapsulated in each NPB507 was then determined. The SAP was established in the range of 8.5–12.5% from the total encapsulated, and the encapsulation efficacy (EE%) varied depending on the antigen (60–90%), which may be attributed to the peptide amino acid sequences. These data are in agreement with other studies that encapsulated tumor antigens in PLGA NPs [30,31]. The protein loading% was calculated as the amount of protein in the NPs divided by the total weight of the particles × 100% (refer to Table S2 for details). As mentioned in the Materials and Methods, Section 4, NPctrl was added to DCs at the same quantity as NPB057 (refer to Section 4.2.4. for more information about the peptide sequences).

The final purpose was to activate T cells against the selected neoantigen, for which the process to be followed is very well described. Once the NP-entrapped neoantigens are taken up by DCs, they transform into mature DCs (mDCs), allowing them to present the antigen. During this process, they overexpress the surface markers HLA-DR, CD80, CD83, and CD86 [32,33].

**Figure 3:**
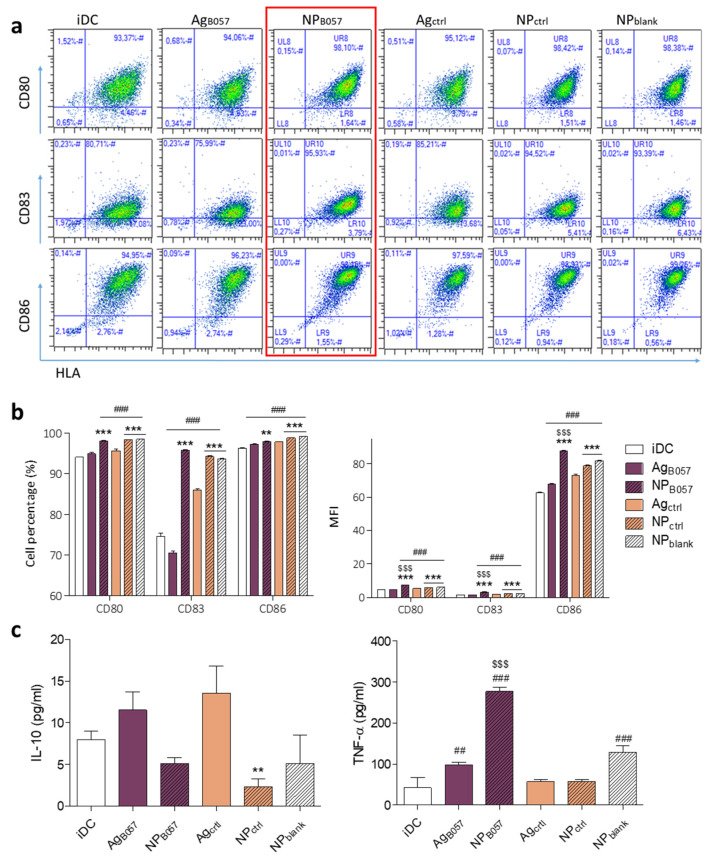
On day 5 after PBMC isolation, iDCs were incubated with free neoantigens and NPs, and maturation was evaluated on day 6. DC maturation was analyzed as CD80^+^-HLA-DR^+^ DCs, CD83^+^-HLA-DR^+^ DCs, and CD86^+^-HLA-DR^+^ DCs. (**a**) Representative flow cytometry plots from DCs after maturation. (**b**) DC maturation markers in cell percentage (%) and mean fluorescence intensity (MFI). (**c**) TNF-α and IL-10 cytokine secretion was measured by ELISA. All samples were analyzed in triplicate (## *p* < 0.01 and ### *p* < 0.001 with regard to iDCs; ** *p* < 0.01 and *** *p* < 0.001 with regard to free antigen; $$$ *p* < 0.001 with regard to the remainder of the groups).

**Figure 4:**
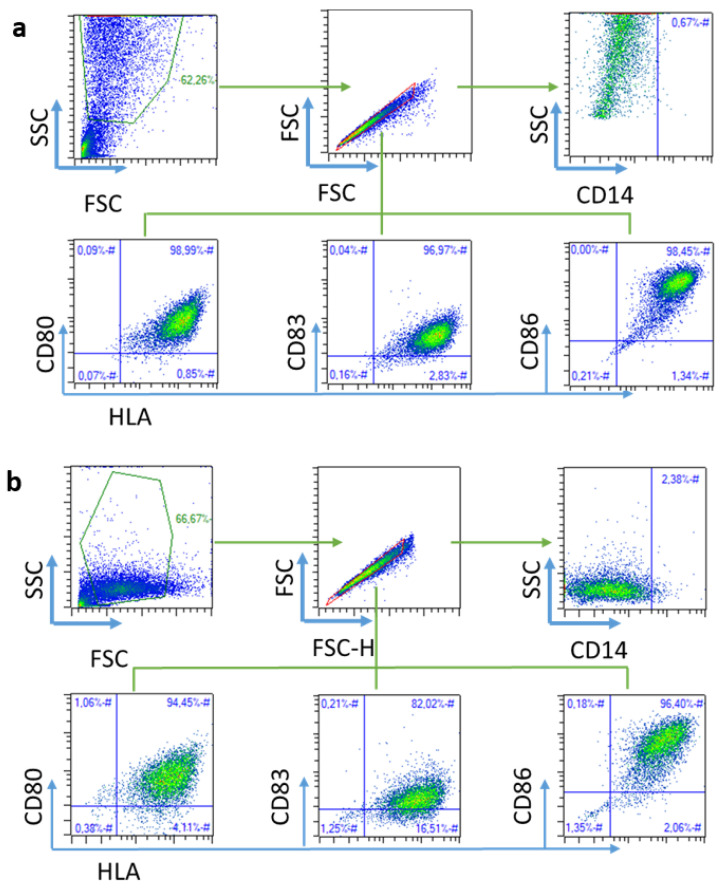
Schematic flow cytometry gating strategy to identify DCs. Two gating strategies were performed for SSC vs. FSC due to the complexity increment when matured with (**a**) NPs compared to when matured with (**b**) free neoantigens, suggesting NP uptake by DCs. Once target cells were characterized by size and granularity, singlets were selected, from which CD14 low cells were defined. DC maturation was determined with an HLA-DR maturation marker vs. CD80, CD83, or CD86.

In order to determine the effects of PEI-coated PLGA NPs on DC maturation, the expression level of maturation markers (HLA-DR, CD80, CD83, and CD86) on immature DCs, DCs treated with free neoantigens (Ag_B057_ and Ag_ctrl_), DCs treated with NP neoantigens (NP_B057_ and NP_ctrl_), and DCs treated with blank NPs (NP_blank_) were assessed after 24 h. DC gating was performed by flow cytometry. As presented in Figure S1, the maturation markers were analyzed based on the forward vs. side scatter chart (FSC vs. SSC) gating. Once the cells were located, CD80, CD83, and CD86 markers were analyzed. The aforementioned markers were plotted against HLA, whereby a higher maturation of the cells displays a graph shifted upwards and to the right. Additionally, the decrease in the percentage of cells expressing the CD14 monocyte marker to confirm the mature DC phenotype (Figure S1) was evaluated. As illustrated in Figure 3b, a greater number of DCs expressed these maturation markers when treated with any NP compared to Immature DCs (iDCs) or DCs stimulated with free neoantigens. These results suggest that the uptake of PEI-coated PLGA NPs (with or without neoantigens) by DCs induced their maturation. Although direct uptake studies were not performed, flow cytometry analysis for FSC vs. SSC demonstrated an increased cell complexity when DCs were cultured with NPs (Figure 4), suggesting the NPs were taken up by the DCs [34]. These results are consistent with those of similar studies conducted using NPs with similar aims [30,35].

It must be mentioned that all three groups treated with NPs (including NP_blank_) had a similar maturation profile when analyzed as a cell percentage (Figure 3b). This indicates that the most important factor triggering maturation is nanoparticulation, in contrast to the mere presence of neoantigens, which only slightly increased DC maturation compared to unstimulated cells. The CD14-positive cell percentage was used to confirm the maturation of DCs, since it decreases with DC maturation status. These results confirm the maturation capacity of the PEI-coated NPs, as they significantly decrease this marker compared to untreated DCs and DCs treated with free neoantigens (Figure 3b). The fact that any NP, and to a lesser extent, any antigen, can trigger DC maturation is due to the unspecific response of DCs to external stimuli [30,31,35].

Apart from the number of cells expressing each marker, the mean fluorescence intensity (MFI) of the maturation markers was also analyzed, indicative of the amount of marker expressed on each cell. Results demonstrated a similar pattern, but higher maturation marker expression was observed for the patient-specific neoantigen NPs (NP_B057_) (Figure 3b).

Regarding cytokine secretion by DCs, TNF-α and IL-10 were analyzed (Figure 3c). Only patient-specific neoantigens were able to significantly increase TNF-α secretion, especially when nanoparticulated. Ag_B057_ and NP_blank_ also have an effect, but to a much lesser extent. TNF-α is an endogenous pyrogen that regulates the immune response and has effects that include the inhibition of tumorigenesis [36]. TNF-α also induces DC migration to lymph nodes, where T cells can be found [37]. Thus, NP_B057_ has the potential to enhance the innate immune response and facilitate a specific response by increasing DC migration and interaction with T cells.

On the other hand, none of the groups significantly increased IL-10 secretion compared to iDCs. This is a positive finding, as this cytokine is related to immunosuppressive mechanisms, T_reg_ activation, and potential inhibition of IFN-γ and IL-2 secretion (T cell activation cytokines) [38,39]. Therefore, neither NPs nor neoantigens provide an immunosuppressive response.

In summary, the results demonstrated that PEI-coated NPs are effective inducers of DC maturation, as they are able to increase maturation markers and trigger correct cytokine secretion.

CD4^+^ cells and CD8^+^ T cell types are required for proper tumor suppression, as CD8^+^ cells (cytotoxic T cells or CTL) need CD4^+^ cells (helper T cells or T_H_) for correct activation [40]. Thus, the effect on T cell proliferation and secretion of cytokines were evaluated.

**Figure 5:**
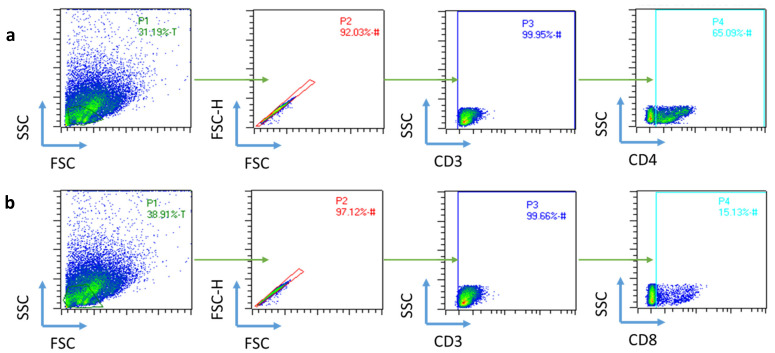
Schematic gating strategy to identify TCs. Cells were characterized by size and granularity, and discriminated by singlets for the selection of (**a**) CD4- or (**b**) CD8-positive T cells. Proliferation analysis (CFSE signal) was conducted from these gating strategies.

In order to determine if free antigen or encapsulated antigen-maturated DCs were able to induce T cell activation, DCs and T cells were co-cultured at a 1:10 ratio. After 5 days, CD4^+^ and CD8^+^ T cell proliferation were analyzed by flow cytometry, and secreted cytokines (IL-2 and IFN-γ) were analyzed by ELISA. In cytometry, T cell gating was performed, locating the cells in SSC vs. FSC gating, followed by a selection of CD3^+^ cells, from which CD4^+^ or CD8^+^ cells were studied (Figure 5).

**Figure 6:**
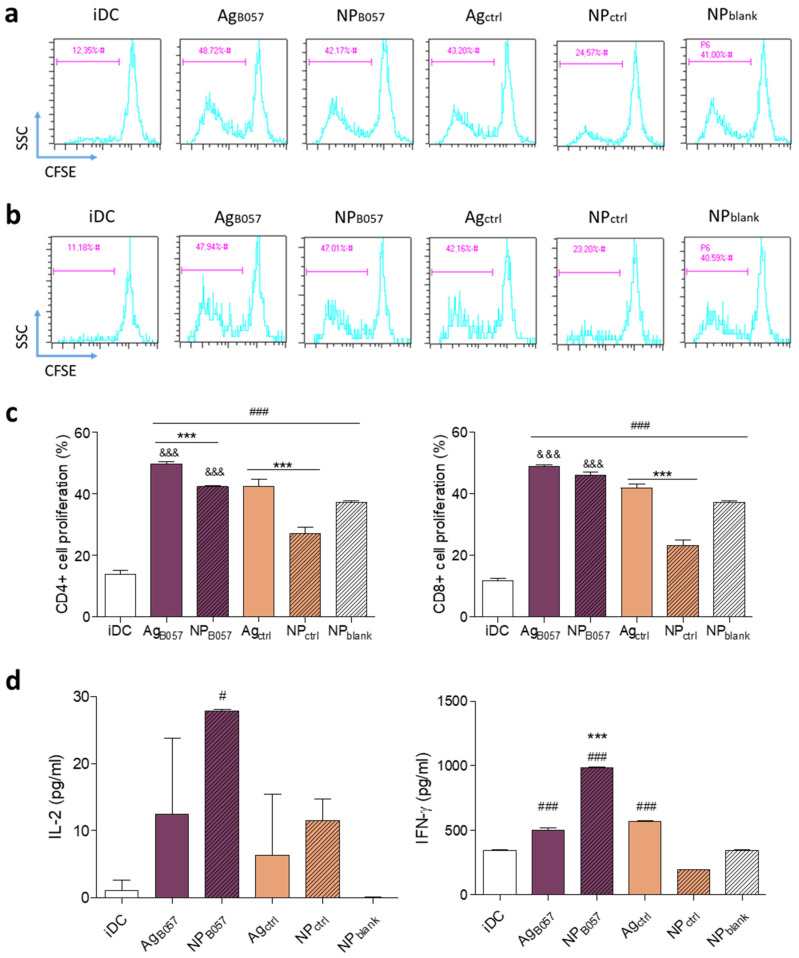
T lymphocyte activation at day 5 after co-culture with autologous DCs at a 1:10 DC:T cell ratio. Representative flow cytometry plots of CFSE-stained (**a**) CD4^+^ and (**b**) CD8^+^ T cells. (**c**) CD4^+^ and CD8^+^ lymphocyte proliferation response in cell percentage (%) induced by DCs. (**d**) IL-2 and IFN-γ cytokine release. Proliferations were analyzed in triplicate and cytokines were analyzed in duplicate (# *p* < 0.05 and ### *p* < 0.001 with regard to iDCs; *** *p* < 0.001 with regard to free antigen; &&& *p* < 0.001 with regard to the homologous control).

T cell proliferation increased with patient-specific neoantigens for both CD4^+^ and CD8^+^ T cells. In the case of CD4^+^ T cells, soluble antigens appeared to induce better proliferation than NPs, and own antigens produced better proliferation than irrelevant ones (Figure 6a,c) [41]. Antigen processing pathways are responsible for the CD4^+^ T cell proliferation rate with free antigens. Dendritic cells process antigens based on their size and shape, so larger particles will be processed as invading pathogens. Therefore, nanoparticles facilitate antigen cross-presentation, leading to CD8^+^ T cell recognition and a cytotoxic response. In contrast, free antigens will not be cross-presented, and CD4^+^ recognition will not occur [42]. On the other hand, both Ag_B057_ and NP_B057_ were able to induce CD8^+^ T cell proliferation, and more significantly than irrelevant antigens (Figure 6b,c). This is of noteworthy importance, as CD8^+^ T cells are the ones with cytotoxic activity.

However, as previously mentioned, the level of T cell activation is not solely determined by their proliferation, but also by their cytokine production, especially by CD4^+^ T cells, which play a crucial role in cancer by aiding CD8^+^ T cells through cytokine release [43].

Figure 6d demonstrates that the release of IL-2, a cytokine that induces lymphocyte growth, proliferation, and survival [44], was significantly increased by NP_B057_. Although T cells produced more IL-2 with NPs than with free antigen, this difference was not statistically significant, likely due to the high variability obtained with free antigens, but it was still significant when compared to iDCs. This finding has been reported in other studies comparing PLGA and other NPs with free antigen [30].

Regarding IFN-γ, NP_B057_ significantly increased cytokine secretion compared to the other groups, indicating greater T cell activation (Figure 6d). These results are consistent with several studies involving PLGA NPs [22,30].

It can be concluded that the T cell response detected in this study is antigen-specific because patient-specific antigens induced a greater T cell activation compared to control groups. Moreover, T cells did not recognize the antigens presented by NPctrl or NPblank, confirming the recognition of the B057 antigen by T cells. It is worth noting that irrelevant antigens have demonstrated a minor capacity to activate T cells, which is in line with other in vitro studies that analyze T cell activation with different antigens. Although T cell activation was better with regard to specific antigens, a slight T cell activation with irrelevant peptides could also be detected in some cases [45,46]. In vivo studies analyzing T cell proliferation have observed greater differences regarding free antigen or NP administration [47,48], as better proliferation was achieved when NPs were used. This could be due to the fact that DCs captured, migrated, and presented the NPs more efficiently than free antigens, leading to a better immune response. In our ex vivo study, as antigens and T cells were in constant contact, this could condition the T cell response and induce proliferation [49,50].

Thus, it has been demonstrated that NP_B057_ is able to induce both CD4^+^ and CD8^+^ T cell proliferation, which is essential for achieving antitumor immunity [40], as both cell types are required for an optimal cytotoxic response [51]. The predominant subtypes of CD4^+^ cells are TH1 and TH2, but TH1 cells are regarded as the most important for cancer immunity. TH1 cells are involved in killing tumor cells by secreting cytokines, including IFN-γ, which enhances the priming and expansion of CD8^+^ cells, among other cytokines and chemokines. Additionally, TH1 cells assist in recruiting natural killer (NK) cells and type I macrophages to tumor sites, leading to tumor eradication [40,43].

Although T cell proliferation was similar to that obtained with Ag_B057_, the encapsulation of the antigen in PEI-coated PLGA NPs led to a higher cytokine release, indicating better T cell activation compared to the free neoantigen, which had less impact on cytokine production.

## 3. Discussion

Recent biotechnological advances have allowed for the genome sequencing of many microorganisms, revolutionizing vaccine development strategies where genomics plays a predominant role. This new approach has been called “Reverse Vaccinology” and begins with the analysis of genome sequences using bioinformatic tools in order to identify the antigens most likely to be vaccine candidates. In particular, next-generation sequencing (NGS) has revolutionized the analysis of genome sequences by allowing the sequencing of exomes, transcriptomes, and even genomes within hours. The investigation of the mutanome is essential, but its complexity is a significant drawback for efficient personalized therapy. In this context, since it is too complex to analyze all of the mutations with current experimental techniques, bioinformatics allows us to address this problem, and is consequently becoming increasingly important in the selection of targets and their prioritization [52].

Nowadays, immunoinformatics emerges as one of the best ways to find potential vaccine candidates against different pathogens, and the selection of the most accurate tools is necessary for predicting and developing potent preventive and therapeutic vaccines [53]. Moreover, due to the lower cost and faster results of in silico/computational studies compared to in vitro/experimental tests, their presence has increased remarkably, not only in vaccine design, but in the experimental designs of biomedical research studies of any kind [54,55,56,57,58,59].

In order to evaluate these tools for personalized vaccine design and test their outcomes experimentally, a comparison among 6048 potential neoantigens and their corresponding peptides without the mutation was performed, using data obtained from six patients suffering from cutaneous melanoma in diverse stages (IB, IIA, IIB, and IIC). In order to carry out this study, both tumor and blood cells were sequenced first, and only mutations that occurred in the tumor were selected. Subsequently, the amino acid sequences surrounding the mutations were identified, which provided us with the set of potential neoantigens. Thereafter, the binding affinity for HLA Classes I and II, and for the Class I immunogenicity, were estimated using the bioinformatic tools provided by IEDB, and in each case, the results of neoantigens and their non-mutated versions were compared.

Our results indicated that, although the number of mutated peptides (i.e., potential neoantigens) presented higher binding affinity in almost every case, the difference was not significant when HLA-I binding affinity or Class I immunogenicity were compared. However, in the case of HLA-II, the number of mutated peptides was significantly higher compared to that of non-mutated peptides. Thus, this study answers, at least partially, the first question, regarding the capacity of these tools to identify potential neoantigens, particularly those that estimate Class II binding affinity. It must be mentioned that, even if the number of potential HLA-II binders was significantly higher in the case of neoantigens, this does not necessarily mean that the binding affinity of mutated peptides is higher. There might be other reasons, such as an overexpression of neoantigens with respect to the non-mutated peptides, etc. There are some tools that estimate the gene expression from the amino acid sequences of proteins, such as the one from [60], but that were hardly applicable to our work. Since this study was planned as a proof of concept of the methodology presented, the specific evaluation of the expression of neoantigens was left for future studies.

In fact, although bioinformatic tools have recently been applied, for instance, to identify B-cell epitopes against human papillomavirus [61] or to predict mutations in mice tumors (where they used MHC Class II binding affinity estimations in order to identify the best epitopes) [62], none so far have performed a one-to-one comparison between mutated and non-mutated estimations to validate the effectiveness of these tools.

Thereafter, in order to be able to evaluate the immune response ex vivo, a vaccine using aggregative functions was developed, instead of using the simpler algorithms used to date [3], which do not always obtain the best combination of the characteristics considered. With our method, a vaccine candidate that optimized seven variables at the same time (i.e., estimated immunogenicity, binding affinity to HLA Class I and Class II, expression levels of the mutations encoding RNA, mutated allele frequencies, hydrophilicity, and relative transcription values) was obtained. This candidate was composed of the best combination of six neoantigens for the evaluated patient. These neoantigens were efficiently encapsulated in PEI-coated PLGA NPs, and were able to induce maturation and activation of the patient’s DCs. Stronger T cell proliferation was detected with patient-specific neoantigens (free and encapsulated) compared to the control groups, especially in CD8^+^ T cells, which are responsible for tumor cell destruction. Moreover, the cytokine release was higher when patient-specific NPs were used, indicating that the overall lymphocyte activation increased with these NPs. In addition, the fact that both arms of the T cell responses studied have been activated suggests that the developed NP could be a promising candidate for anti-melanoma immunotherapy treatment.

Regarding limitations and future work, it is important to note that the studies presented in this work were conducted with single-patient samples, and it would be desirable to carry out more replicates in order to corroborate the results obtained. Additionally, it is worth mentioning that the computational estimations were calculated for each of the 15-amino-acid-long neoantigens, which, in this case, were combined in trios to form 45-amino-acid-long peptides. Therefore, the in silico estimation of the characteristics of the final peptide might differ from the one obtained by the sum of the fragments. However, as explained in the Materials and Methods, Section 4, the characteristics for the most appropriate lengths for each tool were estimated, rather than the longest possible length. For instance, the HLA-I binding tool from IEDB considers peptides of up to 14 amino acids, while the HLA-II tool goes up to 30 amino acids, but not beyond 45 amino acids.

## 4. Materials and Methods

### 4.1. Computational Testing

#### 4.1.1. Sample Acquisition for in Silico Assays

In order to analyze the in silico characteristics of potential neoantigens and test them ex vivo, the first step was to obtain tumor samples from patients with cutaneous melanomas. Tumor biopsies from six patients were obtained and sequenced from Cruces University Hospital and Basurto University Hospital in Spain. Inclusion criteria for patient selection were as follows: (1) a histologically confirmed diagnosis of malignant melanoma; (2) no treatment except for primary surgery (including wide local excision); and (3) no infection, as judged by clinical evaluation and an absence of increased infectious parameters in the blood. Biopsies of melanoma lesions were analyzed by a pathologist.

In order to obtain sufficient mutational diversity, which is an indicator of advanced-stage tumor development, and a heterogeneous sample of different tumor severities for analysis, cases from several cancer stages were selected. The stages of the studied cases, according to the AJCC classification, were as follows: one IB (up to 2 mm thick without ulceration), one IIA (from 1 to 2 mm thick with ulceration or from 2.01 to 4 mm thick without ulceration), two IIB (from 2.01 to 4 mm thick with ulceration or greater than 4 mm thick without ulceration), and two IIC (greater than 4 mm thick with ulceration) [63].

Patient 4 is a woman, 32 years old at diagnosis and without family history of melanoma. Primary melanoma was located on the trunk corresponding to a nodular melanoma in stage IIB, according the American Joint Cancer Committee (AJCC 8th edition). The melanoma tumor was 4.4 mm think (Breslow Index), without ulceration, and BRAF-V600E-positive. Sentinel lymph node detection was not conducted. Patient was untreated, aside from primary surgery. After 54 months of follow-up, lymphatic metastases were detected, and she underwent surgery for metastasis.

Venous blood samples and a primary melanoma biopsy were collected at the hospital one month after the surgery for the primary melanoma, following the protocol established at the Basque Biobank [64]. Genomic DNA was extracted from the buffy coat of peripheral blood samples using the FLEX START DNA Extraction System (Autogen, Holliston, MA, USA) and the FlexiGene DNA Whole Blood Kit (QIAGEN, Hilden, Germany). The primary melanoma tumor was formalin-fixed and embedded in paraffin, and 10 sections of 10 mm thickness were used for DNA isolation using the HigherPurityTM FFPE DNA Isolation Kit (Canvax Biotech, Cordoba, Spain) according to the manufacturer’s instructions.

#### 4.1.2. Detection of Genome Mutations

Subsequently, in order to maximize the cost-effectiveness of our study, the regions with the most variability in this kind of cancer were targeted—in particular, those regions that code for proteins such as BRAF, NRAS, MAP2K1, or MAP2K2 [65,66]. Specifically, AmpliSeq for Illumina Cancer Hotspot Panel v2 panels were used for investigating the hotspot regions of 50 genes known to be associated with cancer.

In order to select only the mutations appearing in the tumor and discard those derived from normal cellular division (and present in non-oncogenic cells), DNA from the blood cells of the same patients and their mutations were also sequenced. Afterwards, only mutation candidates that appeared solely in the tumor were considered.

A set of 50 cancer-associated genes was sequenced from 50 ng of tumor DNA from each patient using the Illumina Cancer HotSpot Panel v2. Library construction was conducted using the ‘Nextera Rapid Capture Exome’ kit, and the size and quality of the purified libraries were checked using an Agilent 2100 Bioanalyzer device. The libraries were then sequenced on an Illumina MiSeq platform with paired-end reads of 150 bp at the Tecnalia Foundation facilities (Genetics lab, Bizkaia, Spain). Following quality control, a range of 300 k reads per library was obtained. The reads were mapped to the GRChr37/hg19 human genome with BWA-MEM, and variants were identified using Cancer Variant Caller 0.9.0.

Specific sequencing of the HLA regions was performed on patients’ blood DNA (400 ng), quantified with Qbit, using the HLA-DNA Sequencing-TruSight HLA v2 Panel, as per the manufacturer’s recommendations. The TruSight HLA v2 workflow amplifies HLA genes in 8 amplicons, each targeting a different HLA region (HLA-A, HLA-B2, HLA-C, DPA1, DPB1, DQA1, DRB.2, and DQB1). The amplified libraries were purified using magnetic beads, and the size and quality of the purified libraries were checked using an Agilent 2100 Bioanalyzer device (Agilent, Santa Clara, CA, USA). The libraries were then sequenced using an Illumina MiSeq platform with paired-end reads of 150 bp at the Genome Analysis Platform at CIC-bioGUNE, Center for Cooperative Research in Bioscience, Bizkaia, Spain. Following quality control, a range of 1.3 to 2.3 million reads per library was obtained, with an average quality over Q35. HLA diversity was characterized using the Illumina TruSight Assign v2 software. The whole genome corresponding to Human Assembly GRCh37/hg19 was downloaded from the UCSC Genome Browser [67].

Since the positions of germline mutations are provided relative to GRCh37/hg19 assembly, and those of tumor mutations are provided relative to GRCh38/hg38 assembly, and since the R package Ensemble used positions relative to GRCh38/hg38, the PyLiftover library [68] was used to convert positions.

#### 4.1.3. Determination of Potential Neoantigens and the Main Characteristics

Once mutations that could potentially generate neoantigens were detected, the next step was to define the length of the neoantigens to be considered, i.e., the number of bases around the mutated base (and its amino acid composition). There are several approaches to this issue; for example, Shahin et al. [3] used 27-mer peptides, while Ott et al. [69] used variable lengths, ranging from 15 to 30.

In our case, for the first analysis, antigens of length 17 were considered, with the mutation in the 9th amino acid. Thus, if a sliding window for peptides with a length of 9 (which has been traditionally used as the standard length for HLA-I restricted T cell antigens [70]) is performed, the mutation will be included in all of them. Thereby, more than one possibility for the estimation of the HLA-II binding affinity was obtained, using sliding windows of length 15, as it is the recommended length for the predicting tool of IEDB. Consequently, the longest potential antigen length considered for computational estimations was also 15 amino acids. Thus, regardless of the processing of the antigens presented by the antigen-presenting cells (APCs), such as dendritic cells or macrophages [71], the mutation will likely be included in one of the presented peptides. For more information about the determination of potential neoantigens, see the Neoantigen Selection Section in the Supplementary Methods.

After identifying the potential neoantigens, the binding affinities of both MHC classes were evaluated. As the HLA complexes are highly polymorphic and vary among individuals, the genes responsible for MHC, located in the 6th chromosome [72], were sequenced, and afterwards, the allele variants for each patient were identified. These variants are presented in Table 1 and Table 2 for Class I and II, respectively.

In order to estimate the MHC Class I and II binding affinities, as well as the Class I immunogenicity predictions, the respective tools from the Immune Epitope Database Analysis Resource [13] were used. For Class I prediction, values for all potential neoantigens ranging from 9 to 14 were calculated, resulting in a total of 5616 potential neoantigens. For Class II prediction, the “default” option was selected, which fixed the antigen length to 15 and resulted in 432 potential neoantigens. Finally, for Class I immunogenicity estimations, strings of length 9, which is the optimal length for the tool, were used.

#### 4.1.4. Statistics

In order to perform the comparisons presented in the Results section, whether the variables were normally distributed was assessed first. Depending on the size of the variable (less than or equal to/greater than 30), either the Shapiro–Wilk or Kolmogorov–Smirnov test, with Matlab 2022 software, was used. Since normality was not rejected in our case, parametric tests were applied in order to compare the data. Two-sample *t*-tests were used to determine if the number of strings that passed the threshold was dependent on whether the string was mutated or not. Thereafter, paired *t*-tests were used to compare those distributions, grouped by patient. The results are reported by the corresponding *p*-values, statistics, and confidence intervals.

### 4.2. Experimental Testing

#### 4.2.1. Sample Acquisition for Ex Vivo Assays

For the second analysis, one of the six patients from the study was selected, namely patient #4. This patient was a woman who was diagnosed with cutaneous melanoma in 1995, at the age of 32, and had no family history of melanoma. The primary tumor was a nodular melanoma located on the trunk, which was classified as stage IIB according to the American Joint Cancer Committee (AJCC 8th edition). The tumor had a Breslow Thickness Index of 4.4 mm, was BRAF-V600E-positive, and demonstrated no ulceration (Figure S2). The patient did not undergo sentinel lymph node detection, and was untreated aside from primary surgery. After 54 months of follow-up, lymphatic metastasis was detected, and the patient underwent an operation.

#### 4.2.2. Estimation of Neoantigens’ Characteristics with Bioinformatic Tools

In order to use the optimal estimations from the IEDB tools, strings of length 15 were taken for this portion of the analysis, with the mutation in the 8th amino acid, since Class I MHC usually accommodates 8–9-mer antigens, while Class II MHC binds to 12–15-mer antigens. Sliding windows of 9 amino acids in length, were used for HLA-I and immunogenicity predictions. The mutation was maintained in the center of the string, following the procedures of previous authors who experimentally tested neoantigen vaccines [3,69].

For every neoantigen n with 15 amino acids, seven values were calculated: an estimation of the Class I immunogenicity [73], an estimation of the binding affinity to HLA-I [74], an estimation of the binding affinity to HLA-II [75], a variant frequency of the mutation, the antigen probability [76], hydrophilicity [77], and TAP proteosome [78]. An extended description of the seven variables can be found in the Supplementary Methods: Estimation of Neoantigens’ Characteristics.

#### 4.2.3. Optimization and Selection of Neoantigens

After the characteristics of the neoantigens were estimated and a 7-tuple

$$v\left(n\right)=\left(Nimm_{i}\left(n\right),NHLAI_{i}\left(n\right),NHLAII_{i}\left(n\right),\;Nvf_{i}\left(n\right),Nap_{i}\left(n\right),\;Nhpl_{i}\left(n\right),NTAP_{i}\left(n\right)\right)$$

was associated to every neoantigen n, the considered function was:

$$f\left(n\right)=0.2\;Nimm_{i}\left(n\right)+0.2\;NHLAI_{i}\left(n\right)+0.2\;NHLAII_{i}\left(n\right)+0.1\;Nvf_{i}\left(n\right)+0.1\;Nap_{i}\left(n\right)+0.1\;Nhpl_{i}\left(n\right)+0.1\;NTAP_{i}\left(n\right).$$

Notice that the most-used characteristics (i.e., the Class I immunogenicity and binding affinity to HLA-I and HLA-II) were weighted doubly with respect to the other three. This consideration was made because those variables have been extensively tested and widely used in the literature for computational vaccine design.

The objective function was $F\left(S\right)=\underset{n\in S}{\sum }f\left(n\right)$, where S is a subset of cardinality 6 of the set N of neoantigens, that is, a set $\left\{n_{1},n_{2},n_{3},n_{4,}n_{5},n_{6}\right\}$ of 6 neoantigens such that

$$F\left(\left\{n_{1},n_{2},n_{3},n_{4,}n_{5},n_{6}\right\}\right)=max_{S\subset N,\;\left|S\right|=6}F\left(S\right),$$

was determined, which is equivalent to taking the 6 neoepitopes with the highest values of $f\left(n\right)$. This solution was grouped into two peptides: the first one was the concatenation of $n_{1},n_{2}$, and $n_{3}$, and the second one was the concatenation of $n_{4,}n_{5}$, and $n_{6}$.

#### 4.2.4. Peptide Synthesis

The neoantigens used in the experiment were custom-made by ChinaPeptides Co. in Shanghai, China. Two neoantigens were selected for the experiment: one from patient #4 (encoded as B-057), and the other from a different melanoma patient (Ag_B057_ and Ag_ctrl_, respectively). As mentioned previously, the antigens were too long, so they were synthesized in two parts: Ag_B057A_ and Ag_B057B_ for the melanoma patient, and Ag_ctrlA_ and Ag_ctrlB_ for the control patient (an irrelevant peptide used as control). All of the synthesized peptides had a length of 45 amino acids.

B057 peptides:

DWLEWLRQLSLELLKFRDQSLSYHHTMVVQIGRFANYFRNLLPSN
MRHSFFSEVNWQDVYRLFMHHVFLEPITCVCSRRFYQFTKLLDSV

Control peptides:

PSLQVITFKQRPRKLSHIRPYMNEIVTLMRFLPQVMPMFLNVIRV
LKCVQFLSQVMPTFLIHCFENVISIMFLVAAGATLERAKTLSPGK

#### 4.2.5. Preparation of PEI-Coated PLGA Nanoparticles (NPs)

PLGA (Resomer-RG503; lactide:glycolide ratio of 50:50; MW 40,600; viscosity 0.41 dL/g; Evonik, Germany), PEI (branched form, molecular weight of 25,000 Da), and polyvinyl alcohol (PVA) were purchased from Sigma Aldrich, Darmstadt, Germany.

PEI-coated PLGA NPs were prepared using the solvent extraction–evaporation double emulsion (*w*/*o*/*w*) method, with encapsulation of melanoma patients’ antigens. Briefly, 50 mg of PLGA and 0.65 mg of PEI were dissolved in 1 mL of dichloromethane (DCM) and emulsified with a 1.25% (*w*/*v*) peptide solution in acetonitrile (ACN):water (50:50) by sonication (30 s using Branson sonifier 250). This emulsion was then mixed with 5 mL of 5% (*w*/*v*) PVA and sonicated for 1 min. The final emulsion was poured into a 2% (*v*/*v*) isopropanol solution (20 mL) and stirred for 2 h to allow solvent evaporation. Finally, the resulting nanoparticles were freeze-dried for 42 h with trehalose used as a cryoprotectant (Lyobeta 15, Telstar, Barcelona, Spain).

#### 4.2.6. Characterization of PEI-Coated PLGA NPs

The size and size distribution of the NPs were analyzed by a dynamic light-scattering technique using an electrophoretic light-scattering photometer. The surface charge of the NPs was determined by measuring the ζ-potential. The Malvern^®^ Zetasizer NanoZS Model ZEN3600 (Malvern Instruments Ltd., Worcestershire, UK) was used for both analyses.

Peptide encapsulation was determined using the microBCA assay within a linear working range of 0–30 μg/mL. (For more information, see the Supplementary Methods: Peptide Encapsulation of PEI-Coated PLGA NPs).

#### 4.2.7. Generation of Monocyte-Derived Dendritic Cells (DCs)

Peripheral blood mononuclear cells (PBMCs) were obtained from melanoma patient B-057. Three EDTA blood tubes were obtained from the patient, and PBMCs were separated by Ficoll-Paque density gradient centrifugation. Monocytes were magnetically isolated using anti-CD14 MicroBeads (MB), and were subsequently cultured with RPMI 1640 prepared medium with 5% inactivated HAS (Human Serum) (Sigma-Aldrich, Darmstadt, Germany), 1% L-glutamine, penicillin/streptomycin (P/S), 400 IU/mL granulocyte-macrophage colony-stimulating factor (GM-CSF), and 200 IU/mL IL-4 (complete medium) for 5 days, at a 2–3 × 10^6^ cell/well concentration in a 6-well plate with 3 ml/well, in order to differentiate immature dendritic cells (iDCs). Medium was replaced on the 3rd day, and on day 5, maturation studies were performed. The remainder of the PBMCs were frozen and stored in N_2_ with 90% HAS and 10% DMSO.

#### 4.2.8. Dendritic Cell Maturation

For DC maturation studies, 50,000 iDCs/well were seeded in a 24-well plate, at a 100,000 cell/mL concentration, in complete medium. Neoantigens were added as free antigens and encapsulated antigens in complete medium, leading to six different experimental conditions: unstimulated iDC, free Ag_B057_, NP_B057_, free Ag_ctrl_ (irrelevant peptide control), NP_ctrl_ (irrelevant encapsulated peptide control), and NP_blank_ (without any antigen). Subsequently, 1 µg Ag_B057_A +1µg Ag_B057_B/50,000 iDC, and an equivalent amount of each peptide entrapped into NP_B057_ A and NP_B057_ B, was added to the corresponding cells. In order to maintain the NP quantity constant in all of the experiments, the same amount of NP_ctrl_A+B and NP_blank_ was used. On day 6, supernatant was obtained for cytokine analysis (IL-10 and TNF-α) and DCs were stained for surface marker analysis (HLA-DR, CD80, CD83, CD86 and CD14). The same experiment was conducted in parallel, and matured DCs were used for T cell experiments.

#### 4.2.9. DC/T Cell Co-Cultures

For autologous cell co-cultures, peripheral blood lymphocytes (PBLs) were obtained from the same samples as DCs and maintained frozen until day 6, as previously explained. Thereafter, the samples were thawed, and lymphocytes were magnetically isolated using anti-CD3 MB and stained with CFSE (5 µM) for posterior proliferation analysis. A 5 mM CFSE solution was prepared according to the manufacturer’s instructions (Sigma-Aldrich, Germany). Subsequently, 1 µL of the CFSE solution was added to 10^6^ cells/mL, maintained at room temperature for 10 min, and then washed with cold medium. CFSE-labeled T cells were co-cultured with matured DCs for 5 days at a DC:T cell ratio of 1:10 in complete medium. On day 11 following blood extraction, supernatant was collected for posterior cytokine analysis, and the surface expression markers CD4 and CD8 were analyzed in T cells by flow cytometry, as well as by CFSE expression.

#### 4.2.10. Flow Cytometry Analysis

For the analysis of DC surface molecules, DCs were stained with APC-conjugated human anti-CD80, PEVio770-CD83, FITC-CD86, PE-CD14, and VioBlue-HLA-DR monoclonal antibodies (Miltenyi Biotech, Bergisch Gladbach, Germany). T cell surface molecules were analyzed with APC-conjugated human anti-CD8 and VioBlue-CD4 (Miltenyi Biotech, Germany), while proliferation was measured with CFSE. In order to avoid unspecific antibody binding, all cytometry samples were previously blocked using FcR Blocking Reagent (Miltenyi Biotech, Germany). Flow cytometry was conducted on a MACSQuant cytometer, and data analysis was performed using the MACSQuantify software v2.13 (Miltenyi Biotech, Germany). The gating strategy is presented in Figure 3 and Figure 5.

#### 4.2.11. ELISA from Supernatants

Supernatants from DCs and DC:T cell co-cultures were collected on days 6 and 11, respectively, and stored at −80 °C until analysis. Two cytokines—IL-10 and TNF-α for DCs, and IL-2 and IFN-γ for T cells—were measured in both supernatants. The concentration of each cytokine was measured in replicates, using specific ELISA kits according to the manufacturer’s instructions (PeproTech, Cranbury, NJ, USA).

#### 4.2.12. Statistics

The results are presented as mean ± standard deviation (SD) for each group. The differences between groups were analyzed using ANOVA, followed by Tukey’s or Bonferroni’s multiple comparison post hoc test, using GraphPad Prism 5.0 software (GraphPad Software, San Diego, CA, USA).

## 5. Conclusions

In this work, a twofold evaluation of computational techniques to detect potential neoantigens has been performed. On the one hand, it was found that the mutated versions have significantly more HLA-II binding affinity according to bioinformatic estimations, and on the other hand, it was confirmed that those techniques were able to select good neoantigens with which to develop a vaccine candidate. The resulting neoantigens, alongside PEI-coated PLGA NPs, generated a strong and specific immune response ex vivo, demonstrating an antitumoral response.

As a result, this study reinforces the use of bioinformatic tools to develop vaccines, using those techniques to obtain new efficient approaches for a rising field, such as vaccine development, which is more necessary than ever.

## Figures and Tables

**Table 1:** HLA-I alleles.

HLA-I	1st Patient		2nd Patient		3rd Patient		4th Patient		5th Patient		6th Patient	
	1st	2nd	1st	2nd	1st	2nd	1st	2nd	1st	2nd	1st	2nd
A	01:01:01	02:01:01	02:01:01	32:01:01	24:02:01	32:01:01	02:01:01	11:01:01	24:02:01	24:02:01	03:01:01	26:01:01
B	08:01:01	44:27:01	35:11:01	51:01:01	07:02:01	51:01:01	27:05:02	40:01:02	35:01:01	40:01:03	07:02:01	18:01:01
C	07:01:01	07:04:01	02:02:02	04:01:01	07:02:01	15:02:01	01:02:01	03:04:01	03:04:01	04:01:01	02:02:02	07:02:01

In the first row, the patient number is presented; in the second, the first or second allele; in the remainder of the rows, the respective Class I HLA alleles: HLA-A, HLA-B, and HLA-C.

**Table 2:** HLA-II alleles.

HLA-II	1st Patient		2nd Patient		3rd Patient		4th Patient		5th Patient		6th Patient	
	1st	2nd	1st	2nd	1st	2nd	1st	2nd	1st	2nd	1st	2nd
DPA1	01:03:01	02:01:01	01:03:01	01:03:01	01:03:01	01:03:01	01:03:01	01:03:01	01:03:01	01:03:01	01:03:01	02:01:01
DPB1	02:01:02	14:01:01	02:01:02	04:01:01	--:01:--	--:01:--	04:01:01	06:01:--	04:01:01	04:01:01	02:01:02	11:01:01
DQA1	01:02:02	01:04:01	01:02:01	05:05:01	01:02:01	01:03:01	03:01:01	04:01:01	01:01:01	04:01:01	01:03:01	05:05:01
DQB1	05:02:01	05:03:01	03:01:01	06:02:01	06:02:01	06:03:01	03:02:01	04:02:01	04:02:01	05:01:01	03:01:01	06:03:01
DRB1	16:01:01	14:54:01	15:01:01	11:04:01	15:01:01	13:01:01	08:01:--	04:04:01	01:01:01	08:02:01	--:--:--	--:--:--
DRB3	02:02:01	--:--:--	02:02:01	--:--:--	01:01:02	--:--:--	--:--:--	--:--:--	--:--:--	--:--:--	01:01:02	01:01:02
DRB4	--:--:--	--:--:--	--:--:--	--:--:--	--:--:--	--:--:--	01:03:01	01:03:01	--:--:--	--:--:--	--:--:--	--:--:--
DRB5	02:02:--	--:--:--	01:01:01	--:--:--	01:01:01	--:--:--	--:--:--	--:--:--	--:--:--	--:--:-	--:--:--	--:--:--

In the first row, the patient number is presented; in the second, the first or second allele; in the remainder of the rows, the respective Class II HLA alleles: HLA-DPA1, HLA-DPB1, HLA-DQA1, HLA-DQB1, HLA-DRB1, HLA-DRB3, HLA-DRB4, and HLA-DRB5.

**Table 3:** Selected neoantigens for the development of the vaccine and the values of the associated variables.

Neoantigen	Immunogenicity	HLA-I	HLA-II	Hydrophilicity	TAP, Proteosome	VaxiJen	Variant Frequency
DWLEWLRQLSLELLK	0.556	0.875	1	0.366	0.722	0.516	0.199
FRDQSLSYHHTMVVQ	0.335	1	0	0.501	0.558	0.453	0.304
IGRFANYFRNLLPSN	0.851	0.665	0.529	0.41	0.567	0.555	0.333
MRHSFFSEVNWQDVY	0.878	0	0.452	0.535	0.72	0.427	0.339
RLFMHHVFLEPITCV	1	0.366	0.432	0	0.603	0	0.762
CSRRFYQFTKLLDSV	0.518	0.562	0.788	0.398	0.608	0.379	0.466

In each column, the normalized values (from 0 to 1) of each variable (Immunogenicity, HLA-I and II binding affinities, Hydrophilicity, TAP proteasome, VaxiJen, and Variant frequency) for the six selected neoantigens. Higher values relate to better probabilities of generating an immune response, binding with molecules, being expressed on the surface, etc.

## Data Availability

The datasets generated and/or analyzed during the current study (the set of potential neoepitopes and the list of estimation from IEDB), as well as the algorithms, are available in the GitHub repository, https://github.com/malainagatsu/Melanoma-aditional-information.git. (accessed on 16 May 2023).

## Supplementary Materials

The supporting information can be downloaded at: https://www.mdpi.com/article/10.3390/ijms24109024/s1.

## Abbreviations

The following abbreviations are used in this manuscript:

ACN	Acetonitrile
Ag	Antigen
APC	Antigen-Presenting Cell
CI	Confidence interval
DC	Dendritic cell
DCM	Dichloromethane
EE	Encapsulation efficacy
GRAVY	Grand average of hydropathicity index
HAS	Human serum
HLA	Human Leucocytic Antigen
IEDB	Immune epitope database
IFN	Interferon
IL	Interleukin
MHC	Major Histocompatibility Complex
NP	Nanoparticle
PBL	Peripheral blood lymphocyte
PBMC	Peripheral blood mononuclear cells
PdI	Polydispersity index
PEI	Polyethylenimine
PLGA	Polyethylenimine-coated L-lactic-co-glycolic acid
SAP	Surface Absorbed Protein
SD	Standard deviation
TAP	Transporter associated with antigen processing
