# Supplementary material for: Computational and Experimental Evaluation of the Immune Response of Neoantigens for Personalized Vaccine Design
Source: Int J Mol Sci. 2023 May 19;24(10):9024. doi: 10.3390/ijms24109024 (PMC10219310; doi:10.3390/ijms24109024)
Supplement: Supplementary file 1 [file ijms-24-09024-s001.zip › ijms-2355837-supplementary.pdf]

Article

Computational and Experimental Evaluation of the Immune Response of Neoantigens for Personalized Vaccine Design

Iker Malaina [1,*,†], Lorena Gonzalez-Melero [2,3,†], Luis Martínez [1,4,*], Aiala Salvador [2,3,5], Ana Sanchez-Diez [6,7], Aintzane Asumendi [7,8], Javier Margareto [9], Jose Carrasco-Pujante [1,4], Leire Legarreta [1,4], María Asunción García [1,4], Martín Blas Pérez-Pinilla [1,4], Rosa Izu [6,7], Ildefonso Martínez de la Fuente [1,4,10], Manoli Igartua [2,3,5], Santos Alonso [11], Rosa Maria Hernandez [2,3,5] and María Dolores Boyano [7,8]

[1] Department of Mathematics, Faculty of Science and Technology, University of the Basque Country (UPV/EHU), 48940 Leioa, Spain
[2] NanoBioCel Research Group, Laboratory of Pharmaceutics, School of Pharmacy, University of the Basque Country (UPV/EHU), 01006 Vitoria-Gasteiz, Spain; rosa.hernandez@ehu.eus (R.M.H.)
[3] Bioaraba, NanoBioCel Research Group, 01009 Vitoria-Gasteiz, Spain
[4] Luis Martínez, Basque Center for Applied Mathematics BCAM, 48009 Bilbao, Spain
[5] Biomedical Research Networking Centre in Bioengineering, Biomaterials and Nanomedicine (CIBER-BBN). Institute of Health Carlos III, 28029 Madrid, Spain
[6] Department of Dermatology, Basurto University Hospital, 48013 Bilbao, Spain
[7] Biocruces Bizkaia Health Research Institute, 48903 Barakaldo, Spain; lola.boyano@ehu.eus (M.D.B.)
[8] Department of Cell Biology and Histology, Faculty of Medicine and Nursing, University of the Basque Country (UPV/EHU), 48940 Leioa, Spain
[9] Technological Services Division, Health and Quality of Life, TECNALIA, 01510 Miñano, Spain
[10] CEBAS-CSIC Institute, Department of Nutrition, 30100 Murcia, Spain
[11] Department of Genetics, Physical Anthropology and Animal Physiology, Faculty of Science and Technology, University of the Basque Country (UPV/EHU), 48940 Leioa, Spain; santos.alonso@ehu.eus
* Correspondence: iker.malaina@ehu.eus (I.M.); luis.martinez@ehu.eus (L.M.); Tel.: +34-946-01-7908 (I.M.)
† These authors contributed equally to this work.

Supplementary Materials

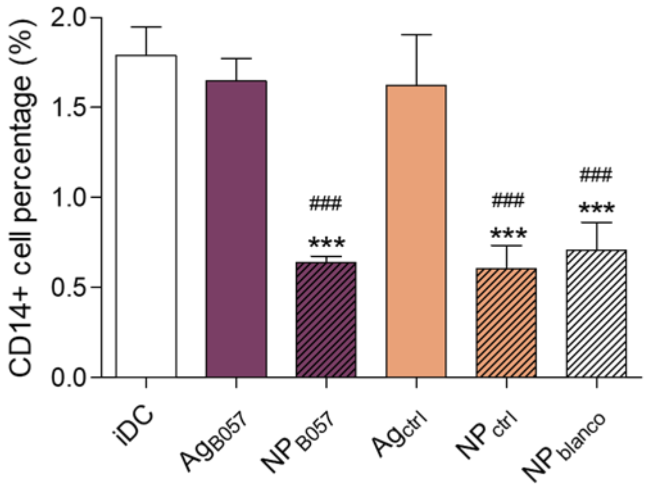

Figure S1. CD14 expressing DCs after incubation with free neoantigens and NPs. CD14 marker is representative of iDCs remaining in the sample, as it decreases with DC maturation. More DCs downregulated CD14 when treated with any NP compared to unstimulated DCs (iDC) or DCs stimulated with free neoantigens. These results suggest that the uptake of PEI-coated PLGA NPs (with or without neoantigens) by DCs induced their maturation.

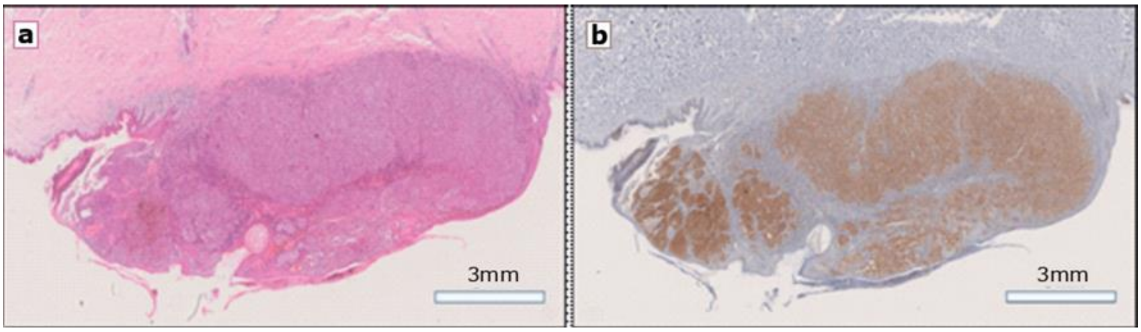

Figure S2. Immunohistochemistry staining of sections of paraffin-embedded biopsy for Hematoxylin-Eosin (**a**) and BRAF V600E (VE1 antibody) contrasted with Hematoxylin (**b**). The formalin fixed paraffin embedded (FFPE) biopsy sections (3 mm) were placed on 3-aminopropyletxylene-covered slides. Subsequently, sections were stained with mouse monoclonal antibody against BRAF V600E (1/100 dilution; clone VE1) following the Ventana Medical Systems' protocol and as previously described [9]. Briefly, staining was performed on a Ventana BenchMark Ultra (Ventana Medical Systems, Inc., Tucson, AZ, USA). The staining protocol included the use of Cell Conditioning 1 for 64 minutes; pre-peroxidase inhibition with 3% hydrogen peroxide for 10 minutes at 37°C and primary antibody incubation for 70 minutes. Amplification kit was applied for 4 minutes at 37°C to increase the signal intensity. The OptiView DAB IHC Detection kit was used to detect BRAF V600E protein expression. Tissues were counterstained with hematoxylin for 16 minutes and Bluing Reagent for 4 minutes. Then, stained sections were scanned by NanoZoomer S210 Digital slide scanner (Hamamatsu Photonics, Japan).

Table S1. Values of all the variables calculated for the potential 15aa length neoantigens, for the 4th patient.

Neoepitope	Immunogenicity	HLA-I	HLA-II	Hydrophilicity - I	TAP, proteosome	VaxiJen	Variant frequency	Combined
**PHCDTLHVLIRDYRE**	0.943	0	0	0.561	0.831	0.49	0.466	0.423
**DWLEWLRQLSLELLK**	0.556	0.875	1	0.366	0.722	0.516	0.199	0.667
**FRDQSLSYHHTMVVQ**	0.335	1	0	0.501	0.558	0.453	0.304	0.449
**NMGNLPLNEFYPAVS**	0.869	0.137	0.207	0.348	0.579	0	0.236	0.359
**IGRFANYFRNLLPSN**	0.851	0.665	0.529	0.41	0.567	0.555	0.333	0.596
**MRHSFFSEVNWQDVY**	0.878	0	0.452	0.535	0.72	0.427	0.339	0.468
**ATAPGAGYYEDTILK**	0.924	0	0	0.398	0.609	0.511	0.476	0.384
**RLRHFPCENVNYGYQ**	0.823	0	0	0.716	0.707	0	0.466	0.354
**GNLGLEGNCTVPPNV**	0.759	0	0	0.33	0.268	0	0.466	0.258
**LRTSTIMIDYNPNYC**	0.704	0	0.236	0.412	0.679	0	0.296	0.327
**HLTDPSQHLVQNCLW**	0.327	0.209	0	0.46	0.753	0.376	0.419	0.308
**QNCLWTLKNLSDAAT**	0.393	0	0.285	0.396	0.336	0.54	0.419	0.305
**SSGAGSPVVAVPTHS**	0.718	0	0.153	0.199	0.376	0	0.37	0.269
**KDSGDYEYAARQATR**	0.835	0	0.08	0.911	0.568	0	0.466	0.378
**NEEDKLKNWEGGLDE**	0.712	0	0.085	1	0.196	1	0.466	0.426
**ENAVGSICYNYLLDV**	0.591	0	0	0.156	1	0.435	0.454	0.323
**VFSHDPLLLGSSSFP**	0.351	0.135	0.08	0.14	0.228	0	0.932	0.243
**RGEGLLPIRWMAPES**	0.99	0	0.26	0.451	0.4	0	0.441	0.379
**APFASSIESHNMTLR**	0.422	0.395	0	0.38	0.509	0.524	0.466	0.351
**TRPFSEALMMGLLTN**	0.305	0.935	0	0.222	0.564	0	0.027	0.329
**GLLTNLAGRELVHMI**	0.899	0.416	0.115	0.055	0.728	0	0	0.364
**QQYECVAKIGEGAYG**	0.778	0	0.137	0.474	0.439	0	0.296	0.304
**SQCLSAPLFVQCGWC**	0.641	0	0	0.032	0.754	0	0.207	0.228
**FCPEPSPLQDPFLPS**	0.443	0	0	0.416	0.208	0.319	0.047	0.188
**GKAGGKPLQSPSQEA**	0	0	0	0.703	0.037	0.346	0.77	0.186
**RELCFPEIRPSFRIR**	0.967	0.144	0	0.501	0.753	0	0.388	0.386
**PVSYSSSSARRPSLD**	0.089	0.379	0	0.59	0.51	0.369	1	0.341
**GGSRNSSFLDHPDER**	0.523	0	0	0.874	0.317	0.606	0.522	0.336
**VCIHKDEMDLLFTDQ**	0.539	0	0	0.357	0.572	0	0.296	0.23
**RLFMHHVFLEPITCV**	1	0.366	0.432	0	0.603	0	0.762	0.496
**GWLIPSNNGKAGKEE**	0.311	0	0	0.686	0.137	0.394	0.493	0.233
**KKIMERDNTAAKTLV**	0.715	0	0	0.547	0.588	0	0.522	0.309
**VSPAAQKVFQPPRSC**	0.387	0.189	0	0.419	0.536	0	0.522	0.263
**SCGTKYEIPIKKKEL**	0.543	0	0.142	0.593	0.55	0	0.522	0.303
**LQRYSEDLTVPLPSE**	0.686	0.487	0	0.538	0.265	0	0.48	0.363
**PSETDGYIAPLTCSP**	0.865	0.288	0	0.405	0.305	0.389	0.234	0.364
**DDGEIKEGTSFAEND**	0.745	0.21	0.088	0.81	0.179	0	0.316	0.339
**ETSIEMEKSELDAQY**	0.341	0	0	0.693	0.433	0	0.661	0.247
**KTLWEDPSIQECYDR**	0.668	0	0.24	0.783	0.583	0	0.248	0.343
**ELKDDDFKRISELGA**	0.656	0	0.164	0.643	0.376	0.849	0.203	0.371
**HLVSRQPMELEVAWT**	0.564	0.403	0.155	0.373	0.551	0	0.187	0.335
**DEGGGYPKPPGAAGG**	0.445	0	0	0.677	0.019	0.69	0.339	0.261
**QPDPKDSYSCLTAAE**	0.119	0	0.102	0.654	0.244	0	0.316	0.166
**ADRGSPAVPGQEDGA**	0.638	0	0	0.638	0.083	0.401	0.248	0.265
**PLALAGPSPPPPPPH**	0.43	0	0	0.453	0	0	0.454	0.177
**CSRRFYQFTKLLDSV**	0.518	0.562	0.788	0.398	0.608	0.379	0.466	0.559

Table S2. Characterization of PEI-coated PLGA NPs.

Formulation	Size (nm)	PdI	Zeta potential (mV)	SAP (%)	EE (%)	Protein loading (%)
NP_{B057} A	278.3 ± 4.7	0.142 ± 0.031	39.0 ± 0.9	8.83 ± 0.28	89.30 ± 11.92	3.68 %
NP_{B057} B	351.4 ± 5.4	0.151 ± 0.055	41.6 ± 0.2	12.69 ± 0.85	62.04 ± 0.26	2.55 %
NP_{blank}	355.9 ± 4.8	0.159 ± 0.020	40.0 ± 0.4	-	-	-
NP_{ctrl} A	335.8 ± 2.3	0.186 ± 0.010	39.4 ± 0.9	17.61 ± 0.97	23.58 ± 2.50	0,97%
NP_{ctrl} B	356.7 ± 3.0	0.177 ± 0.021	41.2 ± 0.1	15.40 ± 2.40	32.61 ± 2.69	1,34%

Particle size, polydispersity index (PdI) and zeta potential were measured to determine particle characteristics. Surface adsorbed protein (SAP), encapsulation efficiency (EE) and NP protein loading was measured to determine antigen encapsulation for B-057 antigen to stablish NP quantity needed for the maturation. All samples were analyzed in triplicate.

Supplementary Methods

Neoantigen Selection

It was important to establish the structure of the neoantigens and the relation of the DNA-mutation and the associated mutation in the peptide. We considered mutations in the amino acid located at the center of the 15mer (or 17mer for the first analysis), with seven non-mutated amino acids before it and another seven after it (or eight in the case of the first analysis). We restricted our attention to mutations in which the mutated peptide is univocally determined by the mutation in the DNA independently of the possible transcripts that can be associated to the DNA-strings and to the peptide. Although in the vast majority of cases there is a unique reading frame, that is, a single reading frame with at least one transcript, this was not always the case, and therefore we considered a priori the three reading frames. For each mutation at the i-th nucleotide position the 15-codons centered at the codon containing the mutation (colored in red) were considered in the three possible reading frames, that is, the three sequences in positions

$$i-21, i-20, i-19, \ldots, i, i+1, i+2, \ldots, i+21, i+22, i+23$$
$$i-22, i-21, i-20, \ldots, i-1, i, i+1, \ldots, i+20, i+21, i+22$$
$$i-23, i-22, i-21, \ldots, i-2, i-1, i, \ldots, i+19, i+20, i+21$$

Each of them corresponds, after transcription and translation, with a peptide of length 15 with a possible mutation at its central amino acid.

Next, for each i and each one of the three reading frames, the transcripts corresponding to the non-mutated sequence were found by using the command “genomeToTranscript” of the R package ensembldb. We searched and analyzed the sequences for which the part of the transcript corresponding to the 51 selected bases was the same for all transcripts, and we found the corresponding translated peptides. These DNA sequences, of course, did not correspond to a unique transcript or a unique protein, but had the particularity that the mutation determined a unique reading frame and a unique 15-peptide, and hence were transcript-independent and protein-independent.

Thus, we selected the neoantigens for which the following conditions hold:

1) The mutation is not non-sense.
2) The corresponding mutation in DNA is non-synonymous.
3) The mutation in DNA is either or a single value mutation or with a multi-nucleotide variant which originate a single amino acid mutation in the center of the neoepitope.
4) None of the associated proteins has any mutation of insertion or deletion type.

Estimation of neoantigens' characteristics

- Estimation of the Class-I immunogenicity. For each of the 7 peptides of length 9 contained in n we calculated the estimation of its immunogenicity using the "T cell Class-I immunogenicity predictor" [74] tool of the IEDB Analysis Resource, and we added these values, obtaining the sequence $\{imm_i\}$ whose length is the number of antigens. After that, we calculated the normalized sequence $\{Nimm_i\}$, where $Nimm_i = \frac{imm_i - m}{M-m}$, being m and M the minimum and maximum, respectively, of $\{imm_i\}$.
- Estimation of the binding affinity to HLA-I. We considered the alleles and the peptides of length 9 contained in n that had a percentile at most 1 in the "Peptide binding to MHC class I molecules" [75] tool of the IEDB Analysis Resource. For each such allele-peptide pair we calculated $-0.5\,p + 1$, where p is the percentile, and we summed up these numbers, obtaining the sequence $\{HLAI_i\}$ whose length is the number of antigens. This way, we gave more weight to the most probable neoantigens. After that, we calculated the normalized sequence $\{NHLAI_i\}$, where $NHLAI_i = \frac{HLAI_i - m}{M-m}$, being m and M the minimum and maximum, respectively, of $\{HLAI_i\}$.
- Estimation of the binding affinity to HLA-II. We considered the alleles that had a percentile at most 10 in the "Peptide binding to MHC class II molecules" [76] tool of the IEDB Analysis Resource for the neoantigen. For each such allele we calculated $-0.05\,p + 1$, where p is the percentile, and we added these numbers, obtaining the sequence $\{HLAII_i\}$ whose length is the number of antigens. Therefore, we gave more weight to the most probable neoantigens. After that, we calculated the normalized sequence $\{NHLAII_i\}$, where $NHLAII_i = \frac{HLAII_i - m}{M-m}$, being m and M the minimum and maximum, respectively, of $\{HLAII_i\}$.
- Variant frequency of the mutation. We calculated the sequence $\{vf_i\}$, whose length is the number of antigens corresponding to the frequency of the mutation in the center of the neoantigen. Thus, we gave more importance to most frequent mutations. After that, we calculated the normalized sequence $\{Nvf_i\}$, where $Nvf_i = \frac{vf_i - m}{M-m}$, being m and M the minimum and maximum, respectively, of $\{vf_i\}$.
- Antigen probability. We calculated the sequence $\{ap_i\}$ whose values were obtained with VaxiJen tool [77]. This tool assigns to each string a probability of being recognized as an antigen, and in particular, a tumor antigen. After that, we calculated the normalized sequence $\{Nap_i\}$, where $Nap_i = \frac{ap_i - m}{M-m}$, being m and M the minimum and maximum, respectively, of $\{ap_i\}$.
- Hydrophilicity. First, we calculated the sequence $\{gr_i\}$ whose length is the number of neoantigens corresponding to the GRAVY index score of the neoantigens obtained with the tool ProtParam [78]. After that, we calculated the normalized sequence $\{Ngr_i\}$, where $Ngr_i = \frac{gr_i - m}{M-m}$, being m and M the minimum and maximum, respectively, of $\{gr_i\}$. Finally, since GRAVY corresponds to values of hydrophobicity, we calculated the sequence $\{Nhpl_i\}$, where $Nhpl_i = 1 - Ngr_i$.
- TAP proteosome. We calculated the sequence $\{TAP_i\}$ whose length is the number of neoantigens whose values were obtained with the tool "Proteasomal cleavage/TAP transport/MHC class I combined predictor" [79] of the IEDB Analysis Resource. After that, we calculated the normalized sequence $\{NTAP_i\}$, where $NTAP_i = \frac{TAP_i - m}{M-m}$, being m and M the minimum and maximum, respectively, of $\{TAP_i\}$.

Peptide encapsulation of PEI-coated PLGA NPs

To determine peptide encapsulation, NPs were disrupted with 0.2N NaOH at 37ºC for 30min under orbital rotation. The SAP (Surface Adsorbed Protein) was determined after NP suspension in ACN-PBS 33% for 30min under orbital rotation at 37ºC. ACN was used for correct peptide dissolution in PBS, and in order to avoid over signalling due to ACN, 33% ACN final concentration was maintained in all wells for microBCA analysis.
